# Hepatosplenomegaly associated with chronic malaria exposure: evidence for a pro-inflammatory mechanism exacerbated by schistosomiasis

**DOI:** 10.1111/j.1365-3024.2008.01078.x

**Published:** 2009-02

**Authors:** S WILSON, F M JONES, J K MWATHA, G KIMANI, M BOOTH, H C KARIUKI, B J VENNERVALD, J H OUMA, E MUCHIRI, D W DUNNE

**Affiliations:** 1 Department of Pathology, University of CambridgeCambridge, UK; 2 Kenya Medical Research InstituteNairobi, Kenya; 3 Division of Vector Borne Diseases, Kenyan Ministry of HealthNairobi, Kenya; 4 DBL, Center for Health Research and Development, Institute for Veterinary Pathobiology, Faculty of Life Sciences, University of CopenhagenDenmark; 5 c/o Kenya Medical Research InstituteNairobi, Kenya

**Keywords:** *co-infection*, *cytokines*, *hepatosplenomegaly*, *human*, *malaria*, Schistosoma mansoni

## Abstract

*In sub-Saharan Africa, chronic hepatosplenomegaly, with palpable firm/hard organ consistency, is common, particularly among school-aged children. This morbidity can be caused by long-term exposure to malaria, or by* Schistosoma mansoni, *and it is exacerbated when these two occur together. Although immunological mechanisms probably underlie the pathogenic process, these mechanisms have not been identified, nor is it known whether the two parasites augment the same mechanisms or induce unrelated processes that nonetheless have additive or synergistic effects. Kenyan primary schoolchildren, living in a malaria/schistosomiasis co-transmission area, participated in cross-sectional parasitological and clinical studies in which circulating immune modulator levels were also measured. Plasma IL-12p70, sTNF-RII, IL-10 and IL-13 levels correlated with relative exposure to malaria, and with hepatosplenomegaly. Soluble-TNF-RII and IL-10 were higher in children infected with*S. mansoni*Hepatosplenomegaly caused by chronic exposure to malaria was clearly associated with increased circulating levels of pro-inflammatory mediators, with higher levels of regulatory modulators, and with tissue repair cytokines, perhaps being required to control the inflammatory response. The higher levels of regulatory modulators amongst*S. mansoni*infected children, compared to those without detectable*S. mansoni *and malarial infections, but exposed to malaria, suggest that*S. mansoni*infection may augment the underlying inflammatory reaction.*

## INTRODUCTION

There are three different levels of immunity to malaria that protect against: severe malaria; mild but symptomatic malaria; or parasitaemia. The latter level of protection being incomplete, with many adults living in endemic areas remaining susceptible to asymptomatic parasitaemia (1). The balance between transmission rates and the development of immunity results in different age-related patterns in the incidence of morbidity. One type of morbidity associated with malaria is hepatomegaly, which in the majority of cases resolves substantially within 2 weeks of treatment (2,3). Splenomegaly caused by acute malaria also usually resolves once the infection has been cleared (4), with regression of splenomegaly occurring with similar kinetics to the resolution of hepatomegaly (2). However, when malaria infections are persistent, or when there is insufficient time between infections for hepatosplenomegaly to fully resolve, chronic hepatosplenomegaly can occur (4).

Age-prevalence curves of hepatomegaly and splenomegaly follow the pattern of the age-prevalence curve for malaria parasitaemia; and hepatosplenomegaly, rather than enlargement of one but not the other organ is common. In hepatosplenomegaly of school-aged children, the liver and spleen are often of a firm to hard consistency and considerably enlarged (5). Early histological studies, based on hepatic needle biopsies, show that among children with firm to hard hepatomegaly, there is coarse fibrosis around the periportal tracts, lymphocyte infiltrations in the hepatic sinusoids and hyperplasia of the Kupffer cells (6,7); indicating that hepatomegaly, like splenomegaly which is attributable to reticuloendothelial and lymphoid hyperplasia (8), is due to chronic inflammation.

As development of immunity to malaria parasitaemia does not develop until late adolescence, school-aged children have ongoing chronic exposure to malaria and often carry low-level, apparently asymptomatic infections, but hepatosplenomegaly is commonly observed in this age group. In areas where *Schistosoma mansoni* is endemic, it is in this same age group that schistosomiasis infection intensity and the prevalence and extent of *S. mansoni* associated hepatosplenomegaly, also peak (9–11). In school-aged children, *S. mansoni*associated hepatosplenomegaly is often seen in the absence of gross, ultrasound detectable, periportal fibrosis (12,13), a type of morbidity more common in adults as it results from prolonged pro-fibrotic responses to infection (14). However, the childhood form of hepatosplenomegaly can also have significant pathological consequences including dilation of portal veins indicating increases in portal pressure, and stunting of growth (13,15). It has been proposed that this form of mainly childhood hepatosplenomegaly is caused by chronic inflammation (11) and it has a similar clinical presentation to hepatosplenomegaly caused by chronic exposure to malaria, in that, in both cases, organs are firm to hard upon palpation (13). The two causes of hepatosplenomegaly were originally thought to have a confounding relationship (9,16), however, there is now evidence that there is an additive or synergistic effect, and that chronic co-exposure to the two parasites can result in both greater prevalence of hepatosplenomegaly (17,18) and a greater extent of organ enlargement (19,20).

As hepatosplenomegaly associated with chronic exposure to malaria and with *S. mansoni*infection may both be caused by inflammation, one explanation for the observed exacerbation of hepatosplenomegaly among school-aged children who are co-exposed, is an augmentation of common underlying immune mechanisms. Two studies report on the influence of *S. haematobium*and malaria co-infection on levels of circulating immune regulators; with levels of IFNγ and sTNF-RII being elevated in co-infected children, either in comparison with children who had blood smear detectable malaria but not detectable *S. haematobium*eggs (21), or in comparison with children who had detectable *S. haematobium*eggs but not blood smear detectable malaria (22). These studies therefore indicate that a pro-inflammatory response, and accompanying down regulatory mechanisms, may be greater in co-infected children than in children infected with either malaria or *S. haematobium*but not the other. If a similar exacerbation of a pro-inflammatory response exists in the relationship between *S. mansoni*and malaria in school-aged children, this may be contributing to hepatosplenomegaly. Here we address this question by examining the levels circulating immune mediators in Kenyan school children in relation to chronic exposure to malaria, active *S. mansoni*infection and clinical presentation with hepatosplenomegaly.

## MATERIALS AND METHODS

### Study population

Children attending two neighbouring primary schools in Makueni District, Kenya participated in a cross-sectional morbidity study. The study area, of 10 by 6 km, has previously been described in detail (23). At the eastern end of the study area, malaria and *S. mansoni*are both transmitted. In the west, malaria but not *S. mansoni*is transmitted. 221 children who attended Yumbuni Primary, in the west of the study area and 228 children, who attended Matangini Primary, in the east of the study area, participated in the study. The age range of the participating children was 4–17 years, with a mean age of 12 years.

### Parasitology and serology

Five stool samples were collected on separate occasions from each participating child. Two 50 mg kato katz slides (24) were prepared from each stool sample for *S. mansoni* egg counts. The egg counts used in the analysis were the average of the ten counts. 8·6% of children attending Yumbuni Primary and 67·1% of children attending Matangini Primary had detectable *S. mansoni*eggs. Amongst those positive for *S. mansoni*, the egg count ranged from 2 to 1356 eggs per gram of stool (epg), with a median infection intensity of 33·7 epg. Blood smears were prepared and examined for malaria parasitaemia at the time of the clinical examinations. Prevalence of microscopy detectable *Plasmodium falciparum*infection was 20·3%. Plasma samples were assayed in triplicate by ELISA for Pfs-IgG3 levels, a marker that accounts both for age-related and geographically-related increases in exposure to malaria, as previously described (23).

### Clinical examination

Each child was examined clinically by palpation for enlarged livers and spleens in the supine position as previously described in detail (13). An organ was considered enlarged if it was palpable more than 2 cm below the costal line. Findings were grouped into four categories for analysis: no organomegaly, firm/hard splenomegaly, firm/hard hepatomegaly or firm/hard hepatosplenomegaly. Children with soft organ enlargement were removed from analysis as these were possibly due to an acute malaria infection (4), which was not the morbidity of interest. No attempt was made to clinically assess the upper margin of either organ and therefore the spans of the organs were not measured. Measurements were taken from the costal margin to the organ edge in order to assess extent of enlargement. Clinical measurements of palpable left liver lobes, taken in the mid-sternal line, were classed into an ordinal variable: no enlargement (0–2 cm), moderate enlargement (3–5 cm) and substantial enlargement (> 5 cm). The spleen was classed as not enlarged if clinical measurements, taken in either the mid-axillary line or the mid-clavicular line, were 0–2 cm; moderately enlarged if either measurement was 2–4 cm and substantially enlarged if either measurement was > 4 cm.

### Cytokine assays

TNFα, IFNγ, IL-4, IL-5, IL-13 and IL-10 were measured by capture ELISA, using matched pair Ab sets (Pharmingen, San Diego, CA), as previously described (25). IL-12p70 and IL-6 were also measured using matched pair antibody sets: capture Ab clones 20C2 and MQ2-13A5 and detecting antibody clones C8·6 and MQ2-39C3, respectively (Pharmingen). IL-12p40 was measured using capture clone C8·3 (Pharmingen) and the same detecting antibody clone as IL-12p70. sTNF-RII was assayed using a matched antibody pair: capture antibody clone 22210 and a polyclonal goat detecting antibody (R&D systems, Minneapolis, USA). Latent TGFβ was acid treated prior to being assayed with 1 µL 1 m HCL per 4 µL sample. Samples were incubated at room temperature for 10 min and neutralized with 1 µL 1·2 m NaOH/0·5 m Hepes/per each 1 µL 1 m HCl added. TGFβ was assayed using a matched antibody pair: capture antibody clone 9016 and a chick IgY polyclonal detecting antibody (R&D Systems). For each cytokine, duplicate plasma samples were assayed in the presence of 5 µL PBS/0·03% Tween 20/10% mouse sera/10% rat sera/10% goat sera (all sera purchased from Serotec, Oxford, UK) to prevent binding of heterophil antibodies. Optical densities were calibrated to standard curves measured by detection of known concentrations of recombinant cytokines, purchased from the same company as the antibody matched pairs, as previously described (25).

### Treatment and ethical considerations

The purpose of the study was carefully explained to members of the communities and informed consent was obtained from the parents or guardians of all children. All children who attended Matangini Primary School, and children who attended Yumbuni Primary School and had detectable *S. mansoni*eggs, were treated with a single dose of praziquantel. Any cases of clinical malaria were treated by a local nurse, as were minor ailments. The study was approved by the Kenya Medical Research Institute national ethical review committee.

### Statistical analysis

Non-parametric techniques were used to analyse the data. Comparisons of levels between two groups was carried out using Mann-Whitney *U*-tests, and between three or more groups using Kruskal–Wallis test, followed by Mann-Whitney *U*-test *post hoc* analysis. Significance levels of *post hoc* analyses are indicated on the relevant figure. Correlation co-efficients were calculated using Spearman's Rank Correlation.

## RESULTS

### Plasma cytokines and parasitological measurements

The levels of none of the plasma cytokines measured were significantly correlated with *S. mansoni*infection intensities (data not shown). The levels of sTNF-RII (*P* = 0·004), IL-10 (*P* = 0·018) and IL-5 (*P* = 0·043) were significant for comparisons between children with neither, one or other single infection or co-infected children (Table 1). *Post hoc* analysis indicated that levels of sTNF-RII (*P* = 0·005), IL-10 (*P* = 0·020), IL-5 (*P* = 0·027) were significantly higher among children who were co-infected with *S. mansoni*and *P. falciparum*, compared with levels among children who had neither infection. Levels of sTNF-RII (*P* = 0·008), IL-10 (*P* = 0·011) and IL-5 (*P* = 0·025) were also significantly higher in children with *S. mansoni*infection without *P. falciparum*infections, compared with children with neither infection. Only plasma levels of sTNF-RII were higher among children who were *P. falciparum*positive but *S. mansoni*negative, compared with children with neither infection. There were no significant differences in the levels of any of the cytokines between children with one or other infection, or co-infected children. Levels of IL-12p70, IL-10, sTNF-RII, IL-4 and IL-13 were correlated with Pfs-IgG3 levels (Table 1). Levels of IL-12p40 were also significantly correlated with Pfs-IgG3 levels; however, the correlation co-efficient was low, indicating that this association was weak.

**Table 1:** Levels of plasma cytokines in relation to parasitological markers

	Sm negative Pf negative	Sm negative Pf positive	Sm positive Pf negative	Sm positive Pf positive	Pfs-IgG3
IL-12p40	143·75	170·08	128·30	150·80	0·099*
	(55.87, 296·98)	(62·63, 380·47)	(40·26, 251·99)	(54·47, 319·85)	
IL-12p70	5·47	2·45	8·76	12·00	0·291***
	(0, 30·85)	(0, 18·97)	(0, 36·76)	(0·79, 112·99)	
TNFα	66·46	60·51	77·58	36·98	0·030
	(6·44, 167·97)	(0, 205·96)	(0, 205·14)	(0, 99·53)	
IFNγ	10·73	12·63	11·02	10·85	0·081
	(4·54, 19·71)	(5·82, 19·59)	(5·42, 18·70)	(6·11, 17·35)	
sTNF-RII	6166	7579*	7357**	7954**	0·316***
	(4394, 8743)	(5131, 10803)	(5240, 9772)	(6432, 10046)	
IL-10	6·76	9·42	9·97*	7·57*	0·357***
	(0·92, 13·83)	(1·99, 18·58)	(3·12, 20·48)	(5·19, 17·77)	
TGFβ	15779	11565	18454	19635	–0·075
	(10124, 24996)	(9107, 20411)	(10731, 31446)	(11459, 29460)	
IL-6	44·57	54·48	50·16	81·96	0·049
	(23·40, 92·14)	(32·13, 116·91)	(16·50, 97·82)	(37·38, 168·69)	
IL-5	4·79	6·09	8·23*	7·69*	0·033
	(0·38, 14·14)	(0·67, 13·14)	(2·34, 15·91)	(2·60, 21·83)	
IL-4	13·81	14·44	16·02	12·72	0·259***
	(7·24, 26·80)	(6·55, 24·37)	(8·97, 28·33)	(8·07, 20·05)	
IL-13	6·67	8·00	9·53	10·65	0·208***
	(0, 19·98)	(0·02, 19·16)	(0·29, 22·80)	(0·21, 40·14)	

Shown are the medians and interquartile ranges of levels of plasma cytokines for children who were negative for both *S. mansoni*and *P. falciparum*infections, infected with one but not the other parasite or were co-infected. Also shown are the Spearman's Rank correlation co-efficients between levels of plasma cytokines and Pfs-IgG3 levels. *Significance levels of comparisons with children with neither infection and of correlations with Pfs-IgG3 levels:

* *P* < 0·05,

** *P* < 0·01,

*** *P* < 0·001.

### Plasma cytokines and presence of organomegaly

**Figure 1:**
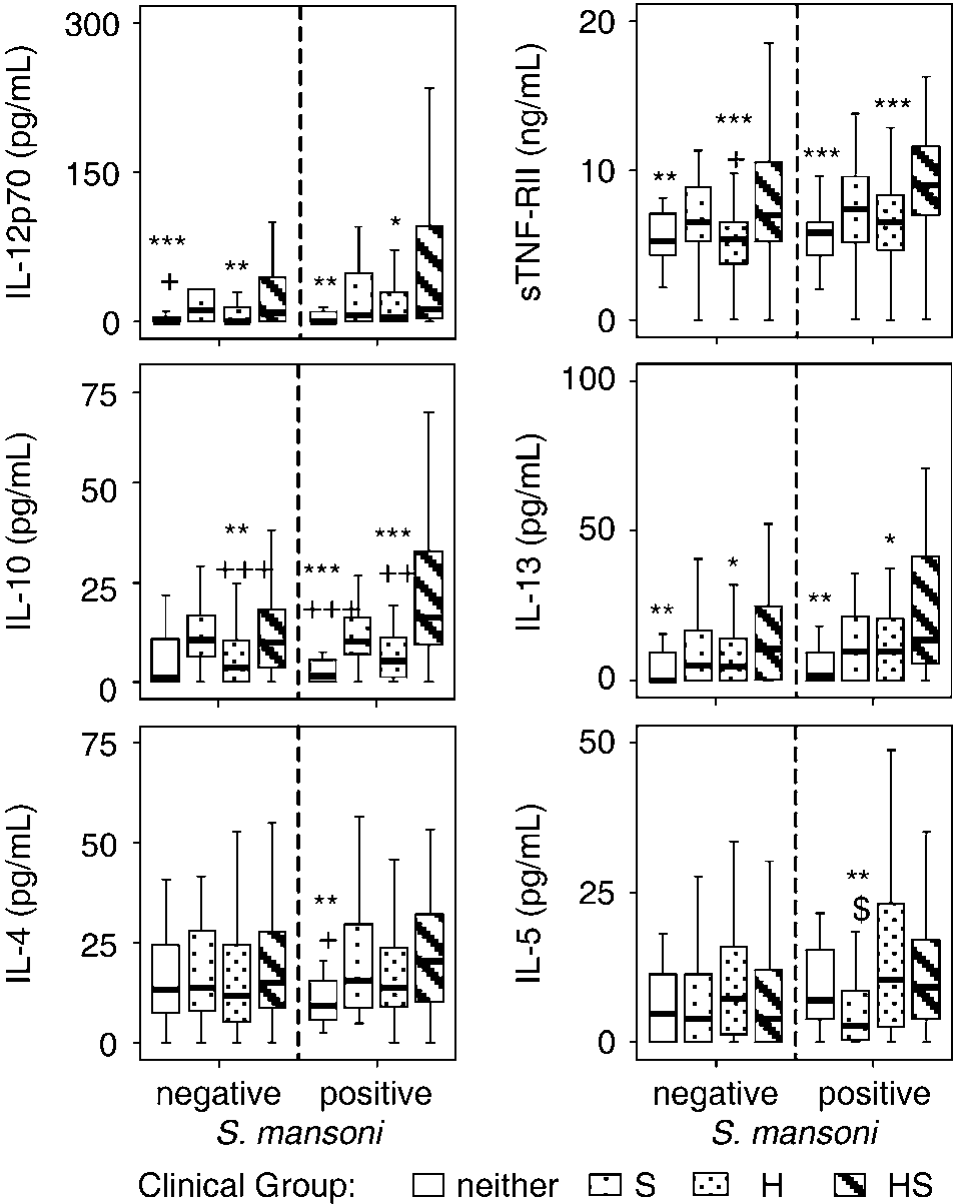
Levels of circulating immune mediators by clinical groupings of organomegaly. Shown are the levels of cytokines which were significantly different between clinical groupings of organomegaly: neither liver nor spleen enlargement, S, spleen enlargement; H, liver enlargement; HS, hepatosplenomegaly. Outliers were removed. *Significantly different from children with hepatosplenomegaly. +Significantly different from children with spleen enlargement. $Significantly different from children with liver enlargement. $/*/+*P* < 0·05, **/++ < 0·001, ***/+++*P* < 0·001.

The associations between plasma cytokines and clinical measurements were analysed separately for children with and without *S. mansoni*infections. The levels of four circulating immune mediators were found to differ significantly between children in the different clinical groups for both *S. mansoni*positive (*Sm*pos) and negative (*Sm*neg) children (Figure 1): IL-12p70 (*Sm*neg: *P* = 0·001; *Sm*pos: *P* = 0·011), IL-10 (*Sm*neg: *P* < 0·001; *Sm*pos: *P* < 0·001), IL-13 (*Sm*neg: *P* = 0·04; *Sm*pos: *P* = 0·018) and sTNF-RII (*Sm*neg: *P* < 0·001; *Sm*pos: *P* < 0·001). Mann-Whitney *post hoc* analysis indicated that those who had hepatosplenomegaly, had significantly higher levels of all four of these cytokines than the children who had no organomegaly, or those with hepatomegaly-only. Levels of circulating IL-10 were significantly higher in children who had splenomegaly-only than in children who had hepatomegaly-only. Levels of sTNF-RII were also significantly higher for children with splenomegaly-only, compared with children with hepatomegaly-only, but this was only significant for children who were *S. mansoni*negative. These four mediators therefore appear to be associated with splenomegaly.

The levels of IL-4 (*P* = 0·024) and IL-5 (*P* = 0·049) were also found to differ significantly between different clinical grouping of organomegaly, but only among children who were *S. mansoni* positive (Figure 1). Mann-Whitney *post hoc* analysis indicated that the level of circulating IL-4 was significantly higher in children who had splenomegaly, compared to children who had no organomegaly. This was irrespective of whether or not the children also had hepatomegaly. Levels of circulating IL-5 were significantly higher in the children who presented with hepatomegaly-only or hepatosplenomegaly, compared with the children who had splenomegaly-only.

### Plasma cytokines and extent of hepatomegaly

**Figure 2:**
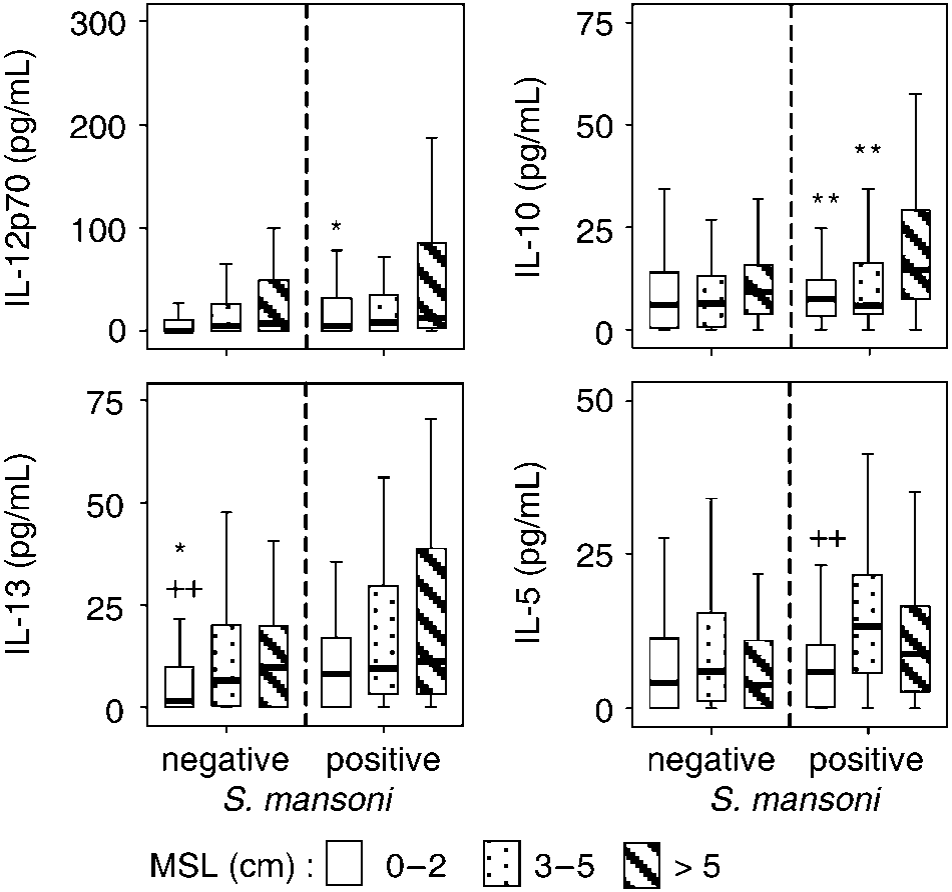
Levels of circulating immune mediators by extent of liver enlargement. Shown are the levels of cytokines that were significantly different between groups representing differing extents of liver enlargement. Outliers were removed. *Significantly different from children with substantial spleen enlargement (> 5 cm). +Significantly different from children with moderate spleen enlargement (3–5 cm). */+*P* < 0·05, **/++*P* < 0·001, ***/+++*P* < 0·001.

For *S. mansoni*negative children, only IL-13 (*P* = 0·013) differed significantly between children with differing extents of liver enlargement. *Post hoc* analysis indicated that the children who had moderate and substantial hepatomegaly had significantly higher levels of circulating IL-13 than the children with no hepatomegaly. This relationship was not significant amongst children who were *S. mansoni*positive, although there was a trend for increased levels of circulating IL-13 in children with moderate and substantial hepatomegaly (Figure 2).

Circulating levels of IL-10 (*P* = 0·002) and IL-5 (*P* = 0·011) differed significantly between children with differing extents of left liver lobe enlargement among those who were *S. mansoni*positive, and IL-12p70 was of borderline significance (*P* = 0·05), so *post hoc* analysis was conducted. IL-12p70 and IL-10 were both linearly related to liver enlargement, with the children who presented with substantial hepatomegaly having significantly higher levels of both cytokines than children who had no hepatomegaly. The children with moderate hepatomegaly also had significantly higher levels of IL-10 compared with those who presented with no hepatomegaly (Figure 2). IL-5 was not linearly related to extent of the left liver lobe enlargement (Figure 2), as *post hoc* analysis indicated that children who had moderate hepatomegaly, but not those who presented with substantial hepatomegaly, had significantly higher levels of circulating IL-5 than those who presented with no hepatomegaly.

### Plasma cytokines and extent of splenomegaly

**Figure 3:**
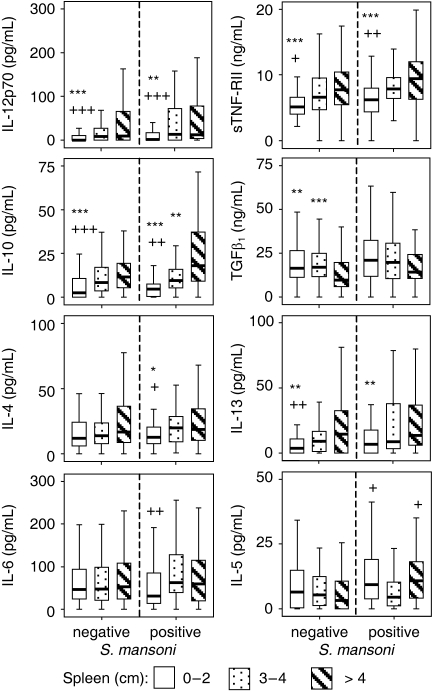
Levels of circulating immune mediators by extent of spleen enlargement. Shown are the levels of cytokines that were significantly different between groups representing differing extents of spleen enlargement. Outliers were removed. *Significantly different from children with substantial spleen enlargement (> 4 cm). +Significantly different from children with moderate spleen enlargement (3–4 cm). */+*P* < 0·05, **/++*P* < 0·001, ***/+++*P* < 0·001.

The levels of the four circulating immune mediators significantly associated with hepatosplenomegaly were all also significantly associated with the extent of spleen enlargement for both *S. mansoni*negative and positive children: IL-12p70 (*Sm*neg: *P* < 0·001; *Sm*pos: *P* = 0·006), IL-10 (*Sm*neg: *P* < 0·001; *Sm*pos: *P* < 0·001), IL-13 (*Sm*neg: *P* = 0·003; *Sm*pos: *P* = 0·017) and sTNF-RII (*Sm*neg: *P* < 0·001; *Sm*pos: *P* < 0·001). For both children who were *S. mansoni*negative and positive, those who presented with moderate or substantial splenomegaly had significantly higher levels of all four cytokines than those with no enlargement of the spleen (Figure 3). For *S. mansoni*positive children, those presenting with substantial splenomegaly also had significantly higher levels of circulating IL-10 than those who presented with moderate splenomegaly. Among children who were *S. mansoni*positive, levels of IL-13 were significantly higher for children with moderate splenomegaly compared with those with no splenomegaly.

For *S. mansoni* negative children, there was a negative relationship between extent of spleen enlargement and levels of TGFβ_1_(*P* < 0·001); those with no enlargement, and those with moderate splenomegaly had significantly higher levels of circulating total TGFβ_1_ than those who presented with substantial splenomegaly. A similar trend was observed for children who were *S. mansoni*positive (Figure 3), but this was not significant (*P* = 0·233).

Three circulating cytokines, IL-6 (*P* = 0·014), IL-4 (*P* = 0·026) and IL-5 (*P* = 0·023), were found to be associated with extent of spleen enlargement of *S. mansoni*positive children but not for *S. mansoni*negative children. Circulating levels of IL-4 were linearly related with the extent of spleen enlargement, as children with either moderate or substantial splenomegaly had significantly higher levels of circulating IL-4 than those who had no splenomegaly. Circulating levels of neither IL-5 nor IL-6 had a linear relationship with the extent of spleen enlargement. Levels of circulating IL-6 were significantly higher in children who presented with moderate enlargement of the spleen, but not those who had substantially enlarged spleens, compared to those who had no enlargement of the spleen (Figure 3). Levels of circulating IL-5 were lowest for children with moderate enlargement of the spleen, as both children with no enlargement of the spleen and those with substantial enlargement of the spleen, had significantly higher levels of circulating of IL-5.

## DISCUSSION

Hepatosplenomegaly among school-aged children has been shown to be exacerbated where malaria and schistosomiasis are co-transmitted (17,19,20), and has pathological consequences, with associated dilation of the portal vein indicating increases in portal pressure, and stunting of growth both being reported (13,15). Hepatosplenomegaly associated with each of these infections is thought to be immune mediated. Here it was shown that chronic exposure to malaria maybe triggering a pro-inflammatory response, as IL-12p70 – a strong inducer of an inflammatory response (26) – was correlated with levels of Pfs-IgG3, a marker of chronic exposure to malaria (23). Levels of IL-12p70 were associated with presentation with hepatosplenomegaly, suggesting that IL-12p70 driven mechanisms could be contributing to the inflammation that is causing organomegaly.

Circulating levels of the classical markers of an inflammatory response, TNFα and IFNγ, may not have been significantly associated with hepatosplenomegaly as they have short-range activity within the tissues of the liver and spleen. It has been shown that Kuppfer cells of children chronically exposed to malaria are increased in number and enlarged (7), and these tissue bound macrophages, if activated, are a possible source of TNFα. The immune system, does though, appear to be mounting a response to control inflammation, as levels of sTNF-RII – a neutralizer of TNFα immunoactivity (27), the shedding of which from human cell lines is induced by TNFα itself (28) – were correlated with Pfs-IgG3. Levels of sTNF-RII were also associated with presentation with hepatosplenomegaly. *In vitro*studies show that control of TNFα production during malaria infections is partly mediated by IL-10 (29,30) and levels of circulating IL-10 were significantly correlated with Pfs-IgG3 levels and presentation with hepatosplenomegaly. Another regulatory immune mediator, TGFβ_1,_ was however, found to be negatively associated with spleen measurements of children who did not have detectable *S. mansoni* eggs. This could be due to thrombocytopenia caused by increased platelet removal by the enlarged spleens, as the levels of TGFβ_1_ substantially reflect the levels released from platelets during venipuncture and the processing of the blood samples (31).

Although IL-12p70 levels were not higher in children with detectable *S. mansoni* eggs, the levels of the immune regulators that control a pro-inflammatory response were higher, as shown by sTNF-RII and IL-10. IL-10 is known to play a role in regulation of human immune responses to schistosomes (32) and previously we have observed that IL-10 levels positively correlate with *S. mansoni*infection intensities (LC Kenty, unpublished results). Others have also reported that levels of circulating IL-10 are higher in *S. mansoni*and *P. falciparum*co-infected adults in comparison with adults solely infected with *P. falciparum* (21). The role of both schistosome infection and malaria infection in the induction of sTNF-RII shedding, is corroborated by the increase in this immune mediator in the plasma of children co-infected with *P. falciparum* and *S. haematobium* in comparison with children who had either of the infections alone (21,22). *Schistosoma mansoni* and malaria associated hepatosplenomegaly in school-aged children in Makueni District, has previously been shown to be associated with increased levels of sTNF-RII (33). The increased production of IL-13, an archetypal Th2 cytokine with pro-fibrotic properties (34), by children with greater exposure to malaria, and its association with hepatosplenomegaly may be due to the necessity to repair tissue damage caused by an inflammatory response.

The circulating levels of IL-6 and IL-5 were found to have statistically significant relationships with hepatosplenomegaly, but in a non-linear fashion. Children who have blood smear detectable malaria have been found to have higher levels of IL-6, compared with children who do not have blood smear detectable malaria infections (35,36) and circulating IL-5 levels were elevated in children who had detectable *S. mansoni* eggs. The associations between hepatosplenomegaly and these two cytokines could therefore reflect the effects of underlying parasitology, rather than a direct association with organomegaly.

Whether the exacerbation of hepatosplenomegaly by co-exposure to *S. mansoni*infection (20) was an amplification of the malaria driven inflammation due to cross-reactivity, or was due to the additive effects of additional anti-schistosome inflammatory responses, cannot be determined from plasma cytokine levels. Exacerbation could be additive due to granulomatous inflammation around trapped eggs, or another mechanism could be that both malaria parasites and adult *S. mansoni*worm make haemozoin (37) as a waste product of their digestion of the host haemoglobin. Haemazoin is phagocytosed by cells of the reticuloendithelial system and can trigger an inflammatory response (38–40). Inflammation around haemozoin loaded cells has been associated with malaria induced hepatomegaly (7). It is not known whether *S. mansoni*derived haemozoin can also drive an inflammatory response. However, it demonstrates a possible simple additive effect of schistosomiasis and malaria that does not necessarily require complex immunological interactions between the host's responses to these two parasites.

To conclude, it is known that long-term exposure to malaria causes chronic enlargement of the liver and the spleen and here the levels of three cytokines, IL-12p70, IL-10 and IL-13, and levels of sTNF-RII were found to be associated with both hepatosplenomegaly and exposure to malaria. The elevated levels of IL-12p70 indicate a pro-inflammatory mechanism, and these are accompanied by higher levels of IL-10 and sTNF-RII, necessary as a down-regulatory mechanism of the inflammatory response. The role of IL-13 is less clear, however, it could be released in order to repair tissue damage caused by an inflammatory response. Children with *S. mansoni* infection, and exposed to malarial infections, had higher circulating levels of IL-10 and sTNF-RII, than those who did not have *S. mansoni* infections, and were exposed to but were negative for malarial infections by microscopy. These elevated levels of the regulatory molecules indicate that the exacerbation of hepatosplenomegaly, and associated pathological consequences, in *S. mansoni*and malaria co-exposure, could be due to an augmentation of the inflammatory response within the liver and spleen.

## References

[1] Marsh K, Kinyanjui S (2006). Immune effector mechanisms in malaria. Parasite Immunol.

[2] Sowunmi A (1996). Hepatomegaly in acute falciparum malaria in children. Trans R Soc Trop Med Hyg.

[3] Sowunmi A, Adedeji AA, Sowunmi CO (2001). Clinical characteristics and disposition kinetics of the hepatomegaly associated with acute, uncomplicated,*Plasmodium falciparum* malaria in children. Ann Trop Med Parasitol.

[4] Greenwood BM (1987). Asymptomatic malaria infections – do they matter?. Parasitol Today.

[5] McGregor IA&, Smith DA (1952). A health, nutrition, and parasitological survey in a rural village (Keneba) in West Kiang, Gambia. Trans R Soc Trop Med Hyg.

[6] Walters JH, Waterlow JC (1954). Fibrosis of the liver in West African children. Spec Rep Series Med Res Counc (G B).

[7] Walters JH, McGregor IA (1960). The mechanism of malarial hepatomegaly and its relationship to hepatic fibrosis. Trans R Soc Trop Med Hyg.

[8] Marsden PD, Hamilton PJ (1969). Splenomegaly in the tropics. Br Med J.

[9] Ongom VL, Bradley DJ (1972). The epidemiology and consequences of *Schistosoma mansoni* infection in West Nile, Uganda. I. Field studies of a community at Panyagoro. Trans R Soc Trop Med Hyg.

[10] Arap Siongok TK, Mahmoud AA, Ouma JH (1976). Morbidity in *Schistosomiasis mansoni* in relation to intensity of infection: study of a community in Machakos, Kenya. Am J Trop Med Hyg.

[11] Gryseels B, Polderman AM (1987). The morbidity of *schistosomiasis mansoni* in Maniema (Zaire). Trans R Soc Trop Med Hyg.

[12] Boisier P, Ramarokoto CE, Ravoniarimbinina P, Rabarijaona L, Ravaoalimalala VE (2001). Geographic differences in hepatosplenic complications of *schistosomiasis mansoni* and explanatory factors of morbidity. Trop Med Int Health.

[13] Vennervald BJ, Kenty L, Butterworth AE (2004). Detailed clinical and ultrasound examination of children and adolescents in a *Schistosoma mansoni*endemic area in Kenya: hepatosplenic disease in the absence of portal fibrosis. Trop Med Int Health.

[14] Booth M, Vennervald BJ, Kabatereine NB (2004). Hepatosplenic morbidity in two neighbouring communities in Uganda with high levels of Schistosoma mansoni infection but very different durations of residence. Trans R Soc Trop Med Hyg.

[15] Corbett EL, Butterworth AE, Fulford AJ, Ouma JH, Sturrock RF (1992). Nutritional status of children with *schistosomiasis mansoni* in two different areas of Machakos District, Kenya. Trans R Soc Trop Med Hyg.

[16] Smith DH, Warren KS, Mahmoud AA (1979). Morbidity in *schistosomiasis mansoni* in relation to intensity of infection: study of a community in Kisumu, Kenya. Am J Trop Med Hyg.

[17] Fulford AJ, Mbugua GG, Ouma JH, Kariuki HC, Sturrock RF, Butterworth AE (1991). Differences in the rate of hepatosplenomegaly due to *Schistosoma mansoni* infection between two areas in Machakos District, Kenya. Trans R Soc Trop Med Hyg.

[18] Whittle H, Gelfand M, Sampson E, Purvis A, Weber M (1969). Enlarged livers and spleens in an area endemic for malaria and schistosomiasis. Trans R Soc Trop Med Hyg.

[19] Booth M, Vennervald BJ, Kenty L (2004). Micro-geographical variation in exposure to *Schistosoma mansoni* and malaria, and exacerbation of splenomegaly in Kenyan school-aged children. BMC Infect Dis.

[20] Wilson S, Vennervald BJ, Kadzo H (2007). Hepatosplenomegaly in Kenyan schoolchildren: exacerbation by concurrent chronic exposure to malaria and *Schistosoma mansoni* infection. Trop Med Int Health.

[21] Diallo TO, Remoue F, Schacht AM (2004). Schistosomiasis co-infection in humans influences inflammatory markers in uncomplicated *Plasmodium falciparum* malaria. Parasite Immunol.

[22] Remoue F, Diallo TO, Angeli V (2003). Malaria co-infection in children influences antibody response to schistosome antigens and inflammatory markers associated with morbidity. Trans R Soc Trop Med Hyg.

[23] Wilson S, Booth M, Jones FM (2007). Age-adjusted *Plasmodium falciparum* antibody levels in school-aged children are a stable marker of microgeographical variations in exposure to *Plasmodium* infection. BMC Infect Dis.

[24] Katz NA, Chaves A, Pellegrino J (1972). A simple device for quantitative stool thick smear technique in *schistosomiasis mansoni*. Rev Inst Med Trop Sao Paulo.

[25] Joseph S, Jones FM, Kimani G (2004). Cytokine production in whole blood cultures from a fishing community in an area of high endemicity for *Schistosoma mansoni*in Uganda: the differential effect of parasite worm and egg antigens. Infect Immun.

[26] Iwasaki A, Medzhitov R (2004). Toll-like receptor control of the adaptive immune responses. Nat Immunol.

[27] Van Zee KJ, Kohno T, Fischer E, Rock CS, Moldawer LL, Lowry SF (1992). Tumor necrosis factor soluble receptors circulate during experimental and clinical inflammation and can protect against excessive tumor necrosis factor α*in vitro* and *in vivo*. Proc Natl Acad Sci USA.

[28] Higuchi M, Aggarwal BB (1994). TNF induces internalization of the p60 receptor and shedding of the p80 receptor. J Immunol.

[29] Ho M, Sexton MM, Tongtawe P, Looareesuwan S, Suntharasamai P, Webster HK (1995). Interleukin-10 inhibits tumor necrosis factor production but not antigen-specific lymphoproliferation in acute *Plasmodium falciparum* malaria. J Infect Dis.

[30] Ho M, Schollaardt T, Snape S, Looareesuwan S, Suntharasamai P, White NJ (1998). Endogenous interleukin-10 modulates proinflammatory response in *Plasmodium falciparum*malaria. J Infect Dis.

[31] Grainger DJ, Mosedale DE, Metcalfe JC (2000). TGF-β in blood: a complex problem. Cytokine Growth Factor Rev.

[32] King CL, Medhat A, Malhotra I (1996). Cytokine control of parasite-specific anergy in human urinary schistosomiasis. IL-10 modulates lymphocyte reactivity. J Immunol.

[33] Mwatha JK, Kimani G, Kamau T (1998). High levels of TNF, soluble TNF receptors, soluble ICAM-1, and IFN-γ, but low levels of IL-5, are associated with hepatosplenic disease in human *schistosomiasis mansoni*. J Immunol.

[34] Gordon S (2003). Alternative activation of macrophages. Nat Rev Immunol.

[35] Kern P, Hemmer CJ, Van Damme J, Gruss HJ, Dietrich M (1989). Elevated tumor necrosis factor α and interleukin-6 serum levels as markers for complicated *Plasmodium falciparum* malaria. Am J Med.

[36] Day NP, Hien TT, Schollaardt T (1999). The prognostic and pathophysiologic role of pro- and anti-inflammatory cytokines in severe malaria. J Infect Dis.

[37] Chen MM, Shi L, Sullivan DJ (2001). Haemoproteus and Schistosoma synthesize heme polymers similar to Plasmodium hemozoin and β-hematin. Mol Biochem Parasitol.

[38] Sherry BA, Alava G, Tracey KJ, Martiney J, Cerami A, Slater AF (1995). Malaria-specific metabolite hemozoin mediates the release of several potent endogenous pyrogens (TNF, MIP-1α, and MIP-1β) *in vitro*, and altered thermoregulation *in vivo*. J Inflamm.

[39] Coban C, Ishii KJ, Sullivan DJ, Kumar N (2002). Purified malaria pigment (hemozoin) enhances dendritic cell maturation and modulates the isotype of antibodies induced by a DNA vaccine. Infect Immun.

[40] Prato M, Giribaldi G, Polimeni M, Gallo V, Arese P (2005). Phagocytosis of hemozoin enhances matrix metalloproteinase-9 activity and TNF-α production in human monocytes: role of matrix metalloproteinases in the pathogenesis of falciparum malaria. J Immunol.

